# Overexpression of a *SDD1-Like* Gene From Wild Tomato Decreases Stomatal Density and Enhances Dehydration Avoidance in Arabidopsis and Cultivated Tomato

**DOI:** 10.3389/fpls.2018.00940

**Published:** 2018-07-04

**Authors:** Samuel Morales-Navarro, Ricardo Pérez-Díaz, Alfonso Ortega, Alberto de Marcos, Montaña Mena, Carmen Fenoll, Enrique González-Villanueva, Simón Ruiz-Lara

**Affiliations:** ^1^Instituto de Ciencias Biológicas, Universidad de Talca, Talca, Chile; ^2^Facultad de Ciencias Ambientales Y Bioquímica, Universidad de Castilla-La Mancha, Toledo, Spain

**Keywords:** stomatal development, *SDD1*, tomato, dehydration avoidance, abiotic stress tolerance

## Abstract

Stomata are microscopic valves formed by two guard cells flanking a pore, which are located on the epidermis of most aerial plant organs and are used for water and gas exchange between the plant and the atmosphere. The number, size and distribution of stomata are set during development in response to changing environmental conditions, allowing plants to minimize the impact of a stressful environment. In Arabidopsis, *STOMATAL DENSITY AND DISTRIBUTION 1* (*AtSDD1*) negatively regulates stomatal density and optimizes transpiration and water use efficiency (WUE). Despite this, little is known about the function of *AtSDD1* orthologs in crop species and their wild stress-tolerant relatives. In this study, *SDD1-like* from the stress-tolerant wild tomato *Solanum chilense* (*SchSDD1-like*) was identified through its close sequence relationship with *SDD1-like* from *Solanum lycopersicum* and *AtSDD1*. Both *Solanum SDD1-like* transcripts accumulated in high levels in young leaves, suggesting that they play a role in early leaf development. Arabidopsis *sdd1-3* plants transformed with *SchSDD1-like* under a constitutive promoter showed a significant reduction in stomatal leaf density compared with untransformed *sdd1-3* plants. Additionally, a leaf dehydration shock test demonstrated that the reduction in stomatal abundance of transgenic plants was sufficient to slow down dehydration. Overexpression of *SchSDD1-like* in cultivated tomato plants decreased the stomatal index and density of the cotyledons and leaves, and resulted in higher dehydration avoidance. Taken together, these results indicate that *SchSDD1-like* functions in a similar manner to *AtSDD1* and suggest that Arabidopsis and tomatoes share this component of the stomatal development pathway that impinges on water status.

## Introduction

Stomata are microscopic pores on the surface of aerial plant organs. These pores function as valves that facilitate gas exchange, opening up for carbon dioxide uptake/oxygen expulsion and closing to limit the excessive loss of water vapor via transpiration (Kearns and Assmann, 1993). Due to this dual function, stomatal physiology and abundance play a key role in the adaptation and acclimation of plants to different habitats with specific environmental conditions (Hetherington and Woodward, 2003). Stomatal density and distribution in individuals of the same species vary between habitats (Tay and Furukawa, 2008; Hamanishi et al., 2012), suggesting that plants can respond to new environmental conditions, such as changes in water status, light intensity and CO_2_ concentration by changing the number and size of the stomata in young leaves (Gray et al., 2000; Xu and Zhou, 2008; Kang et al., 2009; Clauw et al., 2015). The relationship between stomatal abundance/size and stomatal conductance is complex and varies among species. In potato, for instance, a negative correlation has been reported between stomatal density and stomatal conductance (Yan et al., 2012), and many studies have indicated a positive correlation between stomatal density and water use efficiency (WUE) (Sun et al., 2014). Therefore, plants’ ability to adjust the number, size and distribution of the stomata in their leaves may contribute to their fitness under changing environments (Casson and Gray, 2008; Dow et al., 2014). The substantial intraspecific genetic variation found for stomatal abundance traits in wild Arabidopsis accessions (Delgado et al., 2011) further suggests that such traits have been under selective pressure in natural environments. The mechanisms that control stomatal density (number of stomata/mm^2^) during aerial organ development involve the mature leaves, which detect the prevailing environmental conditions and send unknown signals to the nascent leaf primordium, thus modulating stomatal development (Lake et al., 2001).

In *Arabidopsis thaliana*, stomatal development is regulated by a set of genes that ensure an adequate number and distribution of stomata in the mature leaves (reviewed by Blatt et al., 2017; Chater et al., 2017; Qu et al., 2017) and that begin to operate from the first stages of leaf development. Three master genes encoding the bHLH-type transcription factors *SPEECHLESS* (*SPCH*), *MUTE* and *FAMA* are positive regulators of stomatal lineage initiation and progression. These three proteins act sequentially to promote cell division and cell fate transitions during stomata formation. The first asymmetric division of a special protodermal cell called the meristemoid mother cell (MMC) is regulated by SPCH, which initiates a stomatal lineage, giving rise to a larger cell and a small triangular cell called a meristemoid (M); after up to three more asymmetric divisions controlled by SPCH, MUTE determines the transition of the meristemoid into a guard mother cell (GMC), which undergoes a final symmetric division and differentiation regulated by FAMA to generate the two guard cells (GC) constituting the stoma (Bergmann and Sack, 2007; MacAlister et al., 2007; Pillitteri et al., 2007; Pillitteri and Torii, 2007, 2012; Serna, 2009). The bHLHs ICE1/SCRM2 are needed throughout the pathway (Kanaoka et al., 2008), and the two MYB-like factors FLP/MYB88 (Lai et al., 2005), as well as NRPB3 (Chen et al., 2017), participate in guard cell formation. A wealth of negative regulators have been reported for Arabidopsis that restrain stomatal production and ensure correct, functional patterns. Of these are the membrane receptor-like protein of the LRR superfamily lacking the kinase domain TOO MANY MOUTHS (Yang and Sack, 1995) and the LRR-receptor kinases ERECTA (ER) and ERECTA-LIKE 1 and 2 (ERL1 and ERL2) (Shpak et al., 2005). Several intercellular signaling peptides from the EPIDERMAL PATTERNING FACTOR-LIKE (EPFL) family act to produce both a negative (EPF2, EPF1) and positive (STOMAGEN) output (Kondo et al., 2009; Hunt et al., 2010; Sugano et al., 2010). The current view of stomatal development, based on extensive genetic and biochemical evidence from several laboratories, establishes that cell surface receptors (homo and heterodimers involving TMM, ER, ERL1 and ERL2) selectively bind peptides (EPFLs, STOMAGEN and others) to activate a MAPKs (MITOGEN-ACTIVATED PROTEIN KINASES) cascade involving the MAPKKK YDA (YODA) (Gray and Hetherington, 2004), as well as the MAPKKs MKK4/5 and MKK7/9, as well as two MAPKs, MAPK3/6 (Wang et al., 2007). More recently, other membrane receptor kinases have been involved in the activation of the MAPK cascade (SERKs; Meng et al., 2015). Upon activation, the MAPK cascade represses stomatal development by phosphorylating and inactivating the positive factors SPCH (reviewed in Lau and Bergmann, 2012; Torii, 2015) and perhaps MUTE (Popescu et al., 2009). Some of these genes may have additional functions on top of those initially described (de Marcos et al., 2016, 2017). Another component, identified by a loss-of-function mutant with higher density and small stomatal clusters, is the subtilase STOMATAL DENSITY AND DISTRIBUTION 1 (SDD1) (Berger and Altmann, 2000), whose role remains as yet unclear. It was proposed that the SDD1 subtilase was involved in generating active signaling peptides (Berger and Altmann, 2000), but to date no such role has been demonstrated. Genetic analyses indicate that SDD1 acts independently of other signaling components in the stomatal pathway (Pillitteri and Dong, 2013) and, at this moment, the substrates and mechanisms through which SDD1 acts to repress stomatal production are unknown.

Although stomatal patterns and development vary among species, putative orthologs of many of the stomatal development genes described in *A. thaliana* can be identified in different plant lineages (Peterson et al., 2010; MacAlister and Bergmann, 2011; Hamanishi et al., 2012; Villagarcia et al., 2012; Liu et al., 2015; Chater et al., 2017). Despite this, few studies have addressed whether or not such putative orthologs fulfill similar functions to those described in Arabidopsis in other species and these studies concentrate on the stomatal bHLH transcription factors (MacAlister and Bergmann, 2011; Raissig et al., 2017). Of particular interest is the study of genes that regulate stomatal density, because this trait is crucial for photosynthesis and transpiration, and thus for WUE and productivity under drought (Hepworth et al., 2015). In this context, *SDD1* appears as a target gene to be studied in different species. After the pioneering description of *TMM* and *FLP* (Yang and Sack, 1995), *SDD1* was the next gene to be identified as a regulator of proper stomatal patterning in *A. thaliana* (Berger and Altmann, 2000); *AtSDD1* putatively encodes a subtilisin-type proteinase and, until recently, even though another subtilase has been reported to be involved in the response of stomatal development to CO_2_ (Engineer et al., 2014), *SDD1* has been the only one of the 56 *A. thaliana* subtilase-coding genes whose mutations are specifically associated with a phenotypic change in stomatal traits (Rautengarten et al., 2005; Schaller et al., 2011). While loss-of-function *sdd1* mutants show increased stomatal density and frequent pattern errors (Berger and Altmann, 2000), *SDD1* overexpression in a wild type background has been shown to reduce stomatal density (Von Groll et al., 2002). More recently, the negative regulation of WUE and drought tolerance by GTL1 was ascribed to increased stomatal density (SD) due to the repression of *SDD1* transcript accumulation (Yoo et al., 2010). Other studies involving SDD1 and the peptides EPF1 and EPF2 show that decreases in stomatal density reduce water loss with no deleterious effects on photosynthesis (Franks et al., 2015; Wang et al., 2016; Vrablova et al., 2017).

Two closely related tomato species, *Solanum lycopersicum* and *Solanum chilense*, differ from one another in terms of their habitat and abiotic stress response, and also show differences in some stomatal traits (Loyola et al., 2012; Tapia et al., 2015), providing interesting models for investigating the relationship between stomatal development genes and stress responses. The wild tomato *S. chilense* grows at 3,000 m above sea level in the Atacama Desert and has adapted to adverse conditions such as extreme temperatures, drought and salt stress (Warnock, 1991; Tapia et al., 2005). In contrast, *S. lycopersicum* is vulnerable to temperature variations, a relative humidity lower than 60% as well as to soils with poor organic matter content (Loyola et al., 2012). Therefore, the study of the putative orthologs of *AtSDD1* in these species may shed some light on the molecular mechanisms that modulate stomatal development and the adaptation or acclimation of tomato species to changing environmental conditions.

Here, we report on the isolation and functional characterization of the *SchSDD1-like* gene, which shares a sequence identity to a large extent with *SolycSDD1-like* and *AtSDD1*. This gene is highly expressed in young as well as mature *S. chilense* leaves and its overexpression was able to revert the aberrant stomatal pattern and high sensitivity to drought of the Arabidopsis *sdd1-3* loss-of-function mutant. Furthermore, its overexpression in tomato plants reduced the stomatal index and density of cotyledons and leaves, as well as significantly increasing the dehydration avoidance of tomato plants.

## Materials and Methods

### Plant Materials and Growth Conditions

Eight-week-old clonal plants of *Solanum chilense* (Dunal) Reiche and *Solanum lycopersicum* L. cv. Moneymaker were used to study the expression pattern of *SchSDD1-like* and *SolycSDD1-like*, respectively. The plants were grown in pots containing perlite: vermiculite (1:1, v/v) under long-day conditions (16:8 h, light:dark cycle) at 25°C in a greenhouse. *A. thaliana* L. (Heyn), wild type ecotype Columbia-0, mutant line *sdd1-3* (an EMS mutant with a severe *sdd1* phenotype; Delgado et al., 2012) and transgenic *P_RO_35S:SchSDD1-like* plants were grown in a mixture of vermiculite, perlite and peat moss (1:1:1) in a growth chamber at 23°C with a 16:8 h, light:dark photoperiod.

### RNA Isolation and cDNA Synthesis

The total RNA isolation and purification of *S. chilense, S. lycopersicum* and Arabidopsis were performed using the SV Total RNA Isolation System (Promega, Madison, WI, United States). Organ samples were collected and frozen in liquid nitrogen and stored at -80°C until used for RNA isolation. All the RNA isolation for gene expression was done in triplicate for each organ and leaf developmental stage analyzed. RNA integrity was visualized by 2% agarose gel electrophoresis and their concentration and purity (OD260/OD280 ratio > 1.95) were determined with a NanoDrop ND-1000 spectrophotometer (NanoDrop Technologies, Wilmington, DE, United States). All RNA samples were treated with RNase free DNase I (Ambion) to remove traces of contaminant DNA. To prepare first-strand cDNA, 2 μg of total RNA were reverse transcribed in a 20 μL reaction using the oligo d(T) and AffinityScript QPCR cDNA Synthesis Kit (Stratagene, La Jolla, CA, United States) following the manufacturer’s instructions.

### Gene Expression Analyses

Gene transcript levels were measured by quantitative PCR (qPCR) using a Stratagene Mx3000P (Agilent Technologies) system. All reactions were performed using the Brilliant SYBR Green Master Mix (Stratagene) according to the manufacturer’s instructions. For each sample, qPCR reactions were carried out in triplicate (technical repeats) using 10 μL Master Mix, 0.8 μL of 250 nM of each primer, 2 μL of diluted cDNA and nuclease-free water to a final volume of 20 μL. Fluorescence was measured at the end of each amplification cycle. The PCR cycling conditions were as follows: 95°C for 10 min, followed by 40 cycles at 95°C for 15 s, Tm °C according to the specific primer for 20 s and 72°C for 20 s. Melting curve analysis was routinely performed after 40 cycles to verify primer specificity. The 2^-ΔΔC_t_^ method was applied to calculate the fold change in the expression of each gene (Livak and Schmittgen, 2001). *S. lycopersicum GAPDH* mRNA level (accession number U97257, Solyc05g014470) (Orellana et al., 2010) and Arabidopsis *Fbox* (accession number AT5G15710) (Remans et al., 2008) were used as internal normalization controls. The primers used for qPCR analysis were: SolycSDD1q (T°a: 56°C) (*SolycSDD1-like/SchSDD1-like)* F: TTGGAGGAATGGTAATAGGA/R: TGAGAATTGAAGGATCAGTATAG; AtSPCHq (T°a: 58°C) F: TTCTGCACTTAGTTGGCACTCAAT/R: GCTGCTCTTGAAGATTTGGCTCT; SolycSPCHq (T°a: 56°C) F: CATCAGATTCAGCAGACAT/R: CTCTACTAGATTGAGACACTTC; AtFbox (T°a: 58°C) F: TTTCGGCTGAGAGGTTCGAGT /R: GATTCCAAGACGTAAAGCAGATCAA; SolycGAPDHq (T°a: 58°C) F: ACAACTTAACGGCAAATTGACTGG/R: TTACCCTCTGATTCCTCCTTGATTG.

### Identification and Sequence Analysis of *SDD1-Like* Genes

The protein sequence of AtSDD1 (NP_563701), previously reported by Schlüter et al. (2003), was used to find the tomato protein with the highest degree of sequence homology with *AtSDD1* in Solgenomics, a *Solanaceae* family database ^1^, using the BLASTp algorithm. The gene *Solyc09g064490.1* from *Solanum lycopersicum* turned out to be the one coding for the protein in the tomato genome that was most closely related to AtSDD1 at the sequence level. Therefore, the primers used for *SolycSDD1-like* and *SchSDD1-like* amplification were designed according to the nucleotide sequence of Solyc09g064490.1.

To perform a phylogenetic analysis, the deduced full-length amino acid sequences of SolycSDD1-like, SchSDD1-like and other plant subtilase sequences closely related to these proteins were collected and used as templates to perform a multiple sequence alignment using the BioEdit Sequence Alignment Editor v7.0 software (Hall, 1999). The homology of these proteins was analyzed using MEGA 6.0 (Tamura et al., 2013)^2^, and a phylogenetic tree was constructed. The sequence alignment of the characteristic domains of various subtilisin-like serine proteases was performed in strict accordance with the process described by Berger and Altmann (2000).

The GenBank^3^, Solgenomics^4^ or Gramene^5^ accession numbers of the selected SBTs for drawing the phylogenetic tree are described in Supplementary Table S1.

### Cloning of *SchSDD1-Like* and Generation of Overexpressing Transgenic Plants

The full-length coding sequence of *SchSDD1* was amplified from genomic DNA with a PCR reaction using the following pair of primers: SchSDD1-*like*_PBI_-Fw, 5′- TCTAGAATGGGATACAGTACTCAATC-3′ and SDD1*-like*_PBI_-Rv, 5′- GAGCTCTCACTTCATTGATGC -3′. The forward and reverse primers included *Xba*I and *Sac*I restriction sites, respectively. The amplification reaction was carried out with Platinum^®^ Taq DNA Polymerase (Invitrogen, Waltham, MA, United States) and the resulting DNA fragments were cloned into a pGEM-T vector (Promega, Madison, WI, United States) and sequenced. The *SchSDD1* fragment was excised from the pGEM-T by digestion and inserted in the sense orientation into the *Xba*I-*Sac*I sites of pBI121 or the *Bgl*II-*Bst*EII sites of pCAMBIA1303, replacing the β-glucuronidase (GUS) gene, so that the coding region of *SchSDD1* was under the control of the CaMV 35S promoter. The constructs were named 35S:*SchSDD1-like*. The final expression vectors were separately introduced into the *Agrobacterium tumefaciens* strain GV3101 (Sambrook and Russell, 2001). Transformation of the Col-0 wild type and mutant line *sdd1-3* of *A. thaliana* was performed using the floral dip method (Clough and Bent, 1998). Transgenic plants were selected on MS medium containing 50 mg/L kanamycin and supplemented with 500 mg/L augmentin. The generated kanamycin-resistant seedlings were then transferred to a mixture of soil (1:1:1 of vermiculite, perlite and peat moss) for acclimatization and subsequent growth in a greenhouse under controlled conditions. The presence of the transgene was confirmed in the Arabidopsis lines by PCR from gDNA using the forward-specific internal primer for the *SchSDD1-like* gene 5′-GAAAGTTCCTAGCTCTCGGAAGAGTC-3′ and the reverse primer 5′-GCCAAATGTTTGAACGATC-3′, which anneals to the 3′UTR region of the vector. Genomic DNA isolation was performed using the Wizard Genomic DNA Purification Kit (Promega, Madison, WI, United States). Homozygous T2 lines were obtained by self-crossing and were used for the experiments.

Tomato (*S. lycopersicum* cv. Moneymaker) plants carrying the transgene *35S:SchSDD1-like* were obtained by cotyledon transformation, as described by Fillatti et al. (1987). Cotyledon explants from 8-day-old plants were co-cultivated with *A. tumefaciens* carrying the *35S:SchSDD1-like* gene fusion in the binary vector pCAMBIA1303. Calli were transferred onto shoot-inducing medium (sucrose 3% p/v, zeatin 2 mg/L, hygromycin 5 mg/L, augmentin 500 mg/L, 6 g/L phyto agar, 1 ml/L PPM – plant preservative mixture), and the medium was renewed every 20 days. After 5–6 months, the regenerated shoots were excised and transferred to a root-inducing medium for 10 days (sucrose 3% p/v, hygromycin 5 mg/L, augmentin 500 mg/L, 6 g/L phyto agar, 1 ml/L PPM). The resulting antibiotic-resistant plants, which were hemizygous for the transgene (T1), were micropropagated first *in vitro* and later in soil, before being genotyped to confirm the presence of the transgene and used to obtain seeds and perform the experiments.

### Microscopy and Stomatal Density Determination

Stomatal density was determined in Arabidopsis fully expanded first leaves as described (Delgado et al., 2011). Confocal microscopy after propidium iodide staining was performed as described previously (Delgado et al., 2011; Triviño et al., 2013) using a Leica TCS SP2 confocal microscope.

In the tomato plants, stomatal abundance traits were determined in fully expanded organs using dental resin replicas of third leaves, as in Delgado et al. (2011), or in fixed and clarified cotyledons. Fixation was carried out in the excised cotyledons after 12 h in ethanol-acetic acid 9:1 (V/V), followed by an ethanol series (1 h in 90% ethanol; 1 h in 70% ethanol; 1 h in 50% ethanol; 1 h in 30% ethanol; 1 h in distilled H_2_O). The clarification process required incubation in a chloral hydrate/glycerol solution for at least 24 h. The fixed material or dental resin imprint replicas were examined under a Nikon Eclipse 90i upright microscope with DIC optics and a DXM1200C camera for image acquisition. Data were collected from the adaxial or abaxial epidermis, which was achieved by scoring two symmetric areas in the medial region of the organs, each with a surface of 0.4 mm^2^. Micrographs were analyzed with the free software Fiji Image J. At least 10 different plants were counted.

### Dehydration Response Analysis: Water Deficit Shock Treatment

For the measurement of water loss from leaves, rosette leaves from 4-week-old Arabidopsis wild type plants and transgenic T2 lines carrying *35S:SchSDD-like* were excised and placed on weighing paper. In a similar way, 8-week-old fully expanded leaves were detached from wild type and transgenic lines of *S. lycopersicum* and then subjected to the same treatment. All samples were air-dried slowly in a growing chamber under controlled conditions (25°C; 50% relative humidity) and weighed at different time-points, with six replicates per time-point. The water loss percentage at each time-point was calculated as a percentage of the initial weight of the plant tissue.

### Statistical Analysis

The statistical software package GraphPad Prism 6.01^6^ was used for data analysis. An analysis of variance, followed by multiple comparisons with Tukey’s honest significant difference (HSD) mean separation test, was performed to determine the statistical significance of differences in the mean values at *P* ≤ 0.05. For stomatal counting, all measurements were repeated at least three times on two leaves or cotyledons from 10 different plants of different genotypes, and the Student’s *t*-test was used for statistical analysis. The values were computed as the means ± SE of three or more independent experiments.

## Results

### Identification of *SDD1-Like* From *S. chilense*

**FIGURE 1:**
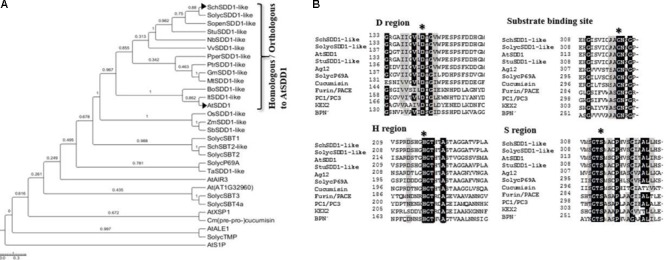
Phylogenetic relationships and sequence alignment of the deduced SchSDD1-like and other homologous subtilisin proteins. **(A)** Phylogenetic relationship between SchSDD1-like and other homologous SBT sequences. The multiple sequence alignments were performed by Clustal W 1.83 and the phylogenetic tree was constructed with Mega 6.0 using the NJ method. The GenBank, Solgenomics and Gramene accession numbers of the SBTs selected to draw the phylogenetic tree were described in the Section “Materials and Methods”. **(B)** Alignment of the sequences of the characteristic domains of various subtilisin-like serine proteases and SchDD1-like. These include the D, H, and S regions, which together form the catalytic triad, and the substrate binding site of different subtilisin-like serine proteases. SDD1 from *A. thaliana* (Berger and Altmann, 2000), Ag12 from *A. glutinosa* (Ribeiro et al., 1995), LeP69 from tomato (Meichtry et al., 1999), cucumisin from melon (Yamagata et al., 1994), FURIN/PACE (Wise et al., 1990) and PC1/PC3 from human (Smeekens and Steiner, 1990), KEX2 from *Saccharomyces cerevisiae* (Mizuno et al., 1988) and subtilisin BPN’ from *B. amyloliquefaciens* (Wells et al., 1983) are shown. Three characteristic catalytic domains (D, H and S regions) and a substrate binding site (N) are marked with an asterisk. Identical residues (>75%) are highlighted in black while similar amino acids are highlighted in gray.

The sequencing of the *Solanum lycopersicum* genome in 2012 (Tomato Genome Consortium, 2012) has allowed the identification of at least 51 sequences that are highly homologous to subtilisin-like protease genes^7^. In potatoes (*S. tuberosum*), the gene *PGSC0003DMG400035001* codes for an SDD1-like subtilase that is probably involved in the modulation of stomatal density, since RNA interference with this sequence resulted in a phenotype similar to *Atsdd1-1* (LMO UId #100293) (BHC, 2004). We used this sequence as a template to search for homologues in the tomato genome using the BLASTp algorithm. The protein putatively encoded by *Solyc09g064490.1.1* (which we named *SolycSDD1-like*) presented the highest identity with this sequence (89%). Using the *SolycSDD1-like* sequence, primers were designed to amplify homologous sequences in the genome of the stress-tolerant wild tomato species *Solanum chilense*. The amplification product obtained was 2,313 bp long and encodes a putative protein of 770 amino acids, which shares 98% identity with SolycSDD1-like. Furthermore, there are no differences in any conserved amino acid residues, and it was therefore named SchSDD1-like. In a similar manner to *A. thaliana SDD1* (Berger and Altmann, 2000), no intron was found in the genomic sequence of *SchSDD1-like*. The predicted protein is related to the family of subtilisin-like serine proteases (subtilases –SBTs), which are commonly found in Archaea, Bacteria and Eukarya and which are more abundant in plants than in other organisms (Rose et al., 2010; Schaller et al., 2011). The predicted SchSDD1-like protein shares 67% of its amino acid sequence identity with the AtSDD1 protein. The phylogenetic analysis, obtained using the protein sequence of SchSDD1-like combined with AtSDD1 and other plant SBTs, revealed a high degree of homology and a close genetic relationship with homologous sequences from different angiosperms (*Solanum tuberosum, Vitis vinifera, Populus trichocarpa*, and *Prunus persica*), which appear to be separated from related monocot SBT proteins (**Figure 1A**). The characteristic catalytic triad present in all subtilases, including the D, H and S regions and a central invariant asparagine residue at the substrate binding site (Dodson and Wlodawer, 1998), were conserved in SolycSDD1-like and SchSDD1-like, and all invariant amino acids were also present (**Figure 1B**). Most plant SBTs are synthesized as pre-pro-proteins and are typically secreted into the apoplast (Schaller et al., 2011; Vartapetian et al., 2011). *In silico* analysis of the SolycSDD1-like and SchSDD1-like deduced protein sequences using the TargetP and Plant-mPLoc online tools (Emanuelsson et al., 2007; Chou and Shen, 2010), indicated the presence of a N-terminal 22-amino-acid signal peptide for targeting to the secretory pathway, similar to that reported for AtSDD1 (Von Groll et al., 2002). Comparison of the sequence with other SBTs showed that the putative start of the mature SolycSDD1-like and SchSDD1-like proteins is in the 111th position of the deduced amino acid sequences (Supplementary Figure S1). Therefore, based on these molecular features shared by both tomato proteins and other characterized SBTs in terms of sequence homology, structural domains and functional sites, we suggest that these predicted proteins are subtilisin-type serine proteases.

### *SDD1-Like* Is Expressed in the Aerial Organs of *S. chilense*

**FIGURE 2:**
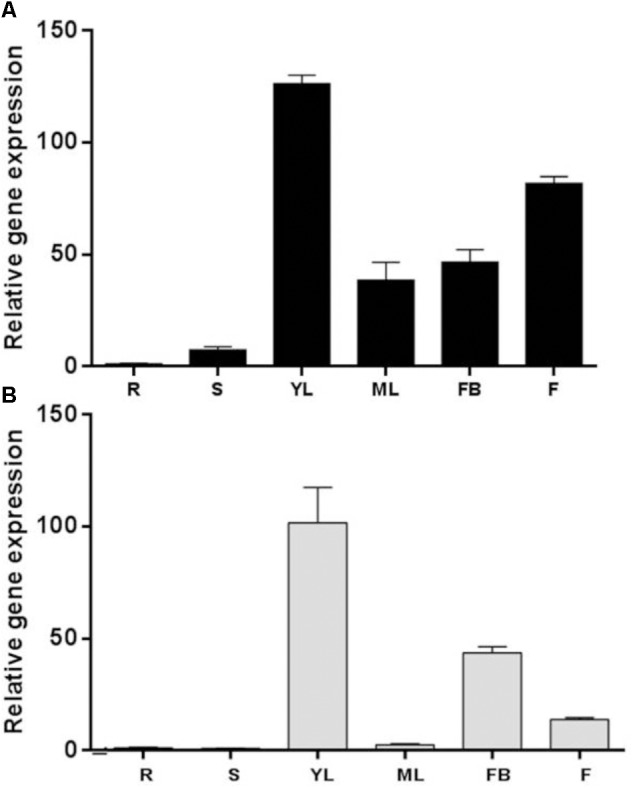
Organ-specific expression pattern of *SDD1-like*. **(A)** Wild tomato *S. chilense*. **(B)** Cultivated tomato *S. lycopersicum*. qPCR analysis of *SchSDD1-like* expression in different organs: *R*, roots; *S*, stems; *YL*, young leaves; *ML*, mature leaves; *FB*, flower buds; *F*, flowers. *SolycGAPDH* was used as an internal control. Data represent mean ± SE (*n* = 3).

In order to investigate the organ-specific expression profiles of *SolycSDD1-like* and *SchSDD1-like*, total RNAs were isolated from the roots, stems, leaves and flowers of *S. lycopersicum* and *S. chilense*, and *SDD1-like* transcripts were evaluated by real-time quantitative PCR. Spatial expression analysis showed that both genes were differentially expressed in the analyzed organs (**Figure 2**). Relatively strong expression levels were detected in the young leaves (defined as small expanding leaves up to 3 cm in length) of both species studied. In contrast, expression was low in the stems and undetectable in the roots. Both species differed in terms of the magnitude of gene expression in mature, fully expanded leaves and in the reproductive organs. In *S. chilense*, expression was relatively high in the mature leaves and flowers (**Figure 2A**), while the level of expression in *S. lycopersicum* organs was almost negligible; only the flower buds accumulated *SDD1-like* transcripts (**Figure 2B**). These data suggest that *SolycSDD1-like* and *SchSDD1-like* participate in processes related with early leaf and flower development in both tomato species. In *S. chilense, SchSDD1-like* may play an additional role in the mature organs. These results also indicate that both genes differ in terms of their gene expression regulatory mechanisms.

### Overexpression of *SchSDD1-Like* in Arabidopsis *sdd1-3* Mutant Restores Wild Type Stomatal Density and Pattern

**FIGURE 3:**
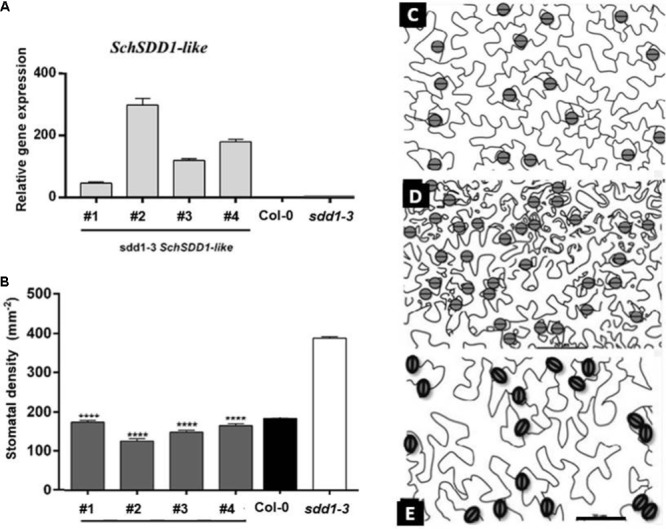
Overexpression of *SchSDD1-like* in the Arabidopsis *sdd1-3* mutant reduced stomatal density in rosette leaves. **(A)** Expression levels of *SchSDD1-like* were measured by qPCR in the leaves of Arabidopsis control plant (Col-0) and in transgenic *sdd1-3* mutant lines. *AtFbox* was used as an internal control. Data represent mean ± SE (*n* = 6). **(B)** Stomatal density in control plants (Col-0), *sdd1-3* and transgenic *sdd1-3* lines. Data represent mean ± SE (*n* = 27). Student’s *t*-test ^∗∗∗∗^*P* ≤ 0.0001. **(C–E)** Representative drawing of leaf abaxial epidermis from Col-0 **(C)**, *sdd1-3* **(D)**, and transgenic *sdd1-3* line #2 **(E)**. The pavement cells are displayed in white and stomata cells are showed in gray.

**FIGURE 4:**
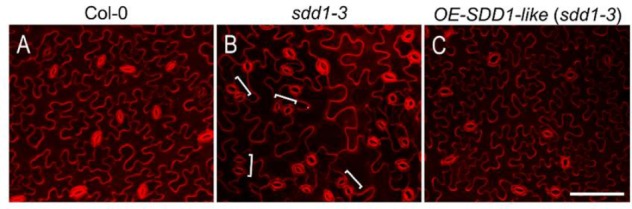
Overexpression of *SchSDD1-like* restores a wild type phenotype in the Arabidopsis *sdd1-3* mutant. Confocal micrographs of abaxial cotyledon epidermis of wild type *Arabidopsis thaliana* (Col-0) **(A)**; *sdd1-3* mutant **(B)**; overexpression lines in the *sdd1-3* genetic background [*OE-SDD1-like* (*sdd1-3*)] **(C)**. Stomatal clusters in *sdd1-3* are shown in brackets. Cotyledons from 20-day- old plants were stained with propidium iodide. Bar: 50 μm.

The *sdd1-1* mutant allele of Arabidopsis has a phenotype characterized by increased stomatal density and a disturbed distribution pattern, with frequent small stomatal clusters (Berger and Altmann, 2000). Therefore, we used the severe *sdd1-3* loss-of-function allele (Delgado et al., 2012), which has a similar stomatal phenotype, to investigate whether *SchSDD1-like* is involved in the regulation of stomatal abundance and pattern. The *Pro35S:SchSDD1-like* gene fusion was mobilized to the wild type Col-0 accession and to the *sdd1-3* mutant via *A. tumefaciens in planta* transformation (Clough and Bent, 1998). Stably transformed plants (T2) were selected according to their kanamycin resistance and genotyped for transgene presence. Transgene expression was determined by qPCR, and four lines with different levels of *SchSDD1-like* mRNA (**Figure 3A**) were selected for further phenotyping. All tested lines showed a significant reduction in first leaf stomatal density compared to *sdd1-3* plants (**Figures 3B–E**). However, no reduction of stomatal density below wild-type values was observed in the *sdd1-3* lines overexpressing *SchSDD1-like*, with the exception of line #2. In addition, these transgenic plants were inspected by confocal microscopy for mutant or wild type phenotypes (i.e., the presence or absence of *sdd1-*characteristic stomatal clusters) in the abaxial epidermis of the cotyledon (**Figure 4**). These results showed that, together with a significantly lower stomatal density, the transgenic lines lacked the stomatal clusters characteristic of the *sdd1-3* phenotype (**Figure 4B**). Taken together, these results indicate that the overexpression of the *SDD1-like* gene from *S. chilense* can revert the *sdd1-3* epidermal phenotype. *SDD1-like* may therefore regulate the density and distribution of stomata in a similar manner to *AtSDD1* in Arabidopsis, suggesting that Arabidopsis and tomato plants might share a common stomatal development pathway.

### *SchSDD1-Like* Overexpression Enhanced the Dehydration Avoidance of *sdd1-3*

**FIGURE 5:**
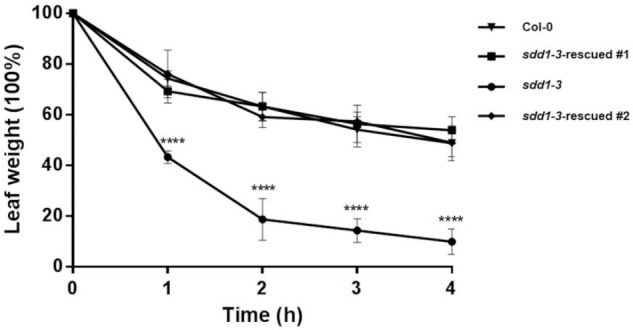
Overexpression of *SchSDD1-like* in Arabidopsis *sdd1-3* mutant increases dehydration avoidance. Water loss in 4-week-old detached leaves at different time-points with three replicates. Three independent experiments were performed with similar results. Data represent mean ± SE (*n* = 6); Student’s *t*-test ^∗∗∗∗^*P* ≤ 0.0001.

To establish whether these *sdd1-3-*rescued lines responded differently to dehydration, as their stomatal abundance was decreased, we performed a leaf dehydration shock assay. The rate of water loss differed significantly between *sdd1-3* and the rescued transgenic plants during the course of the assay (**Figure 5**). In Arabidopsis *sdd1-3* plants, and in accordance with their phenotype showing elevated stomatal density, the leaf fresh weight decreased rapidly compared to the rescued lines and to wild type plants, reaching 10% of the initial weight after 4 h of water deficit shock. On the other hand, we did not observe any important differences in fresh weight between wild type and *sdd1-3-*rescued lines during the stress assay. These observations evidenced that the *SchSDD1-like* gene potentially plays a role in preventing water loss, even in a heterologous species, probably through the regulation of stomatal abundance. Additionally, the results indicate that the stomata of complemented Arabidopsis plants are fully functional in terms of regulating their closure under conditions of water deficit. These results confirm that reduced stomatal density may improve water retention in plants, suggesting its involvement in enhancing tolerance to dehydration (Tricker et al., 2012; Xie et al., 2012).

### *SchSDD1-Like* Overexpression in *Solanum lycopersicum* Reduces Stomatal Abundance and Improves Dehydration Avoidance

**FIGURE 6:**
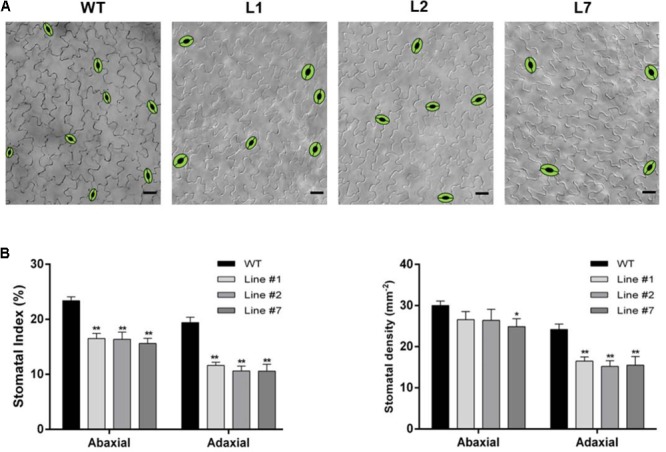
Overexpression of *SchSDD1-like* in tomato plants reduces stomatal index and density in cotyledons. **(A)** DIC micrographs showing representative adaxial epidermal phenotypes in the control (Moneymaker), and three transgenic lines; the stomata have been drawn for clarity. Scale bars: 50 μm **(B)** Adaxial and abaxial stomatal density in cotyledons and stomatal index for the same lines. Data represent mean ± SE (*n* = 27). Asterisks indicate significant differences compared to the control (Student’s *t*-test, ^∗^*P* < 0.05, ^∗∗^*P* < 0.01).

In order to analyze the effects of the ectopic expression of *SchSDD1-like* on stomatal abundance and dehydration avoidance in tomato, we generated transgenic *S. lycopersicum* plants which carried the *Pro35S:SchSDD1-like* gene fusion and selected T1 plants resistant to hygromycin. Three independent transgenic lines showing different levels of relative expression of the transgene (Supplementary Figure S2) were selected. Then, the stomatal density on the abaxial epidermis of fully expanded leaves was scored (Supplementary Figure S3), with the result that, in the three transgenic lines, stomatal density was lower than in non-transgenic tomato leaves. We also measured stomatal traits in the expanded cotyledons (**Figure 6**). The microscopy analysis of cotyledon epidermis showed a significantly reduced stomatal index on the adaxial and abaxial faces in the three transgenic lines in comparison with wild type tomato plants (**Figure 6A**). Stomatal density was also lower on the abaxial and adaxial faces of the cotyledons of the transgenic lines than it was in the wild type plants; however, the statistical significance of these differences was more pronounced for the adaxial side (**Figure 6B**). These results indicate that SchSDD1-like is able to modulate stomatal frequency and distribution in tomato cotyledons. Taken together, these results show that Arabidopsis and tomato plants share a common stomatal development pathway, in which SDD1 acts as a negative regulator in this molecular pathway.

**FIGURE 7:**
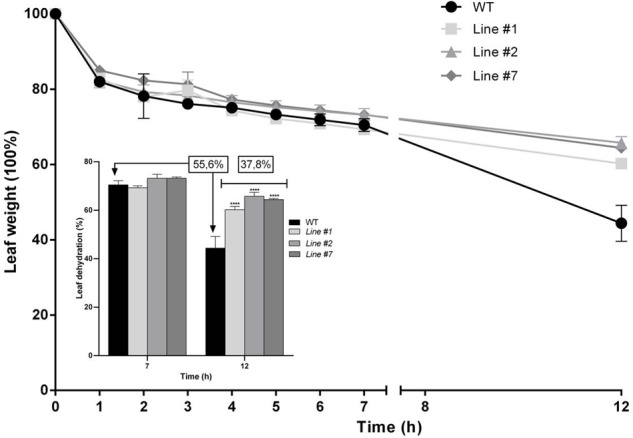
*SchSDD1-like* overexpression in tomato plants increases dehydration avoidance. Water loss in detached leaves of 8-week-old wild type *S. lycopersicum* and transgenic plants was evaluated at different times in three independent experiments and three technical replicates were taken. Data represent mean ± SE (*n* = 6); Student’s *t*-test ^∗∗∗∗^*P* ≤ 0.0001. The insert shows weight loss at seven and 12 h after the onset of dehydration treatment in wild type and transgenic tomato lines.

With the aim of evaluating the role of SchSDD1-like in the responses to water deficit, the 8-week-old fully expanded leaves of wild type and transgenic tomato plants were collected and subjected to a dehydration shock stress assay. The leaves were detached and their water loss rate was determined during the course of 12 h (measured as leaf weight decay). **Figure 7** shows that during the first 7 h of dehydration treatment, no significant differences in water loss were observed between wild type and transgenic tomato plants. However, after 12 h of dehydration treatment, the leaves of transgenic plants showed more improvement in dehydration avoidance than those of wild type plants, because the leaves of plants overexpressing *SchDD1-like* showed an average water loss of 37.8%, while this value was 55.6% for the leaves of wild type plants. During the course of the assay, there were no significant differences in the rate of water loss between the transgenic lines. These results support the idea that a lower stomatal density in tomato leaves controls water loss from the plant, thus improving its response to dehydration.

### *SchSDD1-Like* Modulates the Expression of the Regulatory Master Gene *SPCH* in Arabidopsis

**FIGURE 8:**
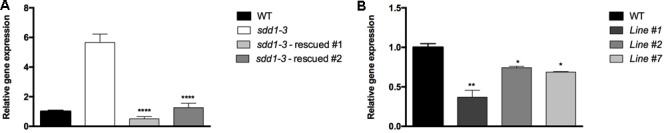
Overexpression of *SchSDD1-like* in Arabidopsis *sdd1-3*-rescued and *S. lycopersicum* transgenic lines affects transcript levels of *AtSPCH* and *SolycSPCH-like*. Expression levels of *AtSPCH* and *SolycSPCH-like* in leaves were measured by qPCR during the early stages of development. **(A)** Wild type Arabidopsis (Col-0), *sdd1-3* mutant and *sdd1-3-*rescued lines. *AtFbox* was used as an internal control. **(B)** Wild type *S. lycopersicum* and *SchSDD1-like* overexpressing lines. Gene expression was normalized against *SolycGAPDH*. Data represent mean ± SE (*n* = 6). Asterisks indicate significant differences when compared to wild type plants (Student’s *t*-test, ^∗^*P* < 0.05, ^∗∗^*P* < 0.01, and ^∗∗∗∗^*P* ≤ 0.0001).

To determine whether or not *SDD1-like* in *S. chilense* functions similarly to the native *AtSDD1* in Arabidopsis, we evaluated *SPCH* gene expression in wild type, *sdd1-3* and *sdd1-3*-rescued lines. *sdd1-3* mutant plants exhibit an elevated level of *SPCH* expression compared to wild type plants (5.5-fold), whereas *sdd1-3*-rescued lines restored transcript abundance to almost wild type levels (**Figure 8A**). Moreover, the overexpression of *SPCH* in *S. lycopersicum* affects the expression of the putative *SolycSPCH-like*, which was significantly down-regulated in all transgenic lines (**Figure 8B**). This fact suggests that *SchSDD1-like* acts like its homologue, AtSDD1, in the same regulatory pathway and that the decreased stomatal density in *sdd1-3*-rescued lines and tomato plants overexpressing *SchSDD1-like* is due to the low expression of the positive regulator *SPCH*.

## Discussion

Arabidopsis plants with different levels of stomatal density imposed by alterations in SDD1 activity also show differences in terms of transpiration and photosynthesis, and therefore have different WUE (Schlüter et al., 2003; Yoo et al., 2010; Lawson et al., 2014). For this reason, *SDD1* has been proposed as a key regulator of WUE through changes in stomatal abundance in leaves. Despite this, little is known about the function of *AtSDD1* orthologs in crop species. However, different stomatal patterns in related *Solanum* species may result, at least in part, from differences in the activity of their *SDD1* orthologs. In an effort to understand the molecular mechanism involved in the regulation of stomatal development in the related species of *S. chilense* and *S. lycopersicum*, we have identified, isolated and functionally characterized the *SchSDD1-like* gene that encodes a putative subtilisin-like serine protease. Furthermore, we have provided evidence of its capacity to regulate stomatal abundance in the epidermis of leaves.

At least 51 putative SBTs sequences have been identified in the *S. lycopersicum* genome, but until now, only a few members of this large gene family have been studied (Tornero et al., 1996; Meichtry et al., 1999; Jordá et al., 2000; Cedzich et al., 2009) and there are no reports of functional involvement for any of them in stomatal development. *SolycSDD1-like* and *SchSDD1-like* are the most closely related tomato genes to the *SDD1* gene from *Solanum tuberosum*. This gene is the only one in the Solanaceae family that has been described as being involved in stomatal abundance, since transgenic potatoes expressing an RNAi construct against the endogenous *StSDD1* gene showed a stomatal phenotype similar to that of the Arabidopsis *sdd1-1* mutant (LMO Record #100293). The phylogenetic analysis grouped these subtilisins from the *Solanum* genus within the clade containing orthologs of the AtSDD1 (**Figure 1**). In addition, the typical domains of subtilisin-type serine proteases were also found in SolycSDD1-like and SchSDD1-like, which contained three characteristic catalytic domains (D, H and S regions) (Polgar, 2005) and the protease associated (PA) domain, which mediates protein–protein interactions between protease and substrate. In the S domain of both SDD1-like proteins, the Ser is inside on a Gly-Thr-Ser-Met-Ala-Cys-Pro motif, which is highly conserved in other members of the subtilase family (Rawlings et al., 2010). Taken together, the results suggest that the proteins encoded by *SolycSDD1-like* and *SchSDD1-like* are similar in structure, and may also be fulfilling equivalent functions to those of a subtilisin-type serine protease in their respective species.

In tomato, leaf stomatal abundance is determined during the plant’s early developmental stages (Gay and Hurd, 1975). Therefore, the high level of *SolycSDD1-like* and *SchSDD1-like* expression that we observed in young leaves seems to coincide with a role in stomatal development, and is similar to the expression levels reported for *AtSDD1* and other orthologous genes like *IiSDD1* and *ZmSDD1* (Xiao et al., 2009; Liu et al., 2015). The transcriptional pattern shown by both *SDD1-like* genes in other organs indicates that the regulation of their expression in the two *Solanum* species is different, and suggests that their functions may also partly differ. Although the high expression level of *SchSDD1-like* in flowers agree with those observed in *AtSDD1*, which apparently does not play a specific role in this organ, it might be an example of gene redundancy. This redundancy cannot be ruled out in flowers, since the repression of *SolycTMP*, which codes for a subtilase related to microsporogenesis and which is highly expressed in flowers, did not trigger phenotypic abnormalities. This suggests its functional redundancy along with other flower subtilases (Riggs et al., 2001). However, since the specific substrates of these proteases have not been identified, it is not possible to determine their function *in planta* in the distinct organs in which they are expressed. On the other hand, the different mechanisms of *SDD1-like* gene regulation between the two tomato species could also be modulated by environmental conditions. In this context, it is tempting to speculate that the increased drought tolerance of *S. chilense* compared to *S. lycopersicum* might be, at least in part, attributed to the different responses of these genes under this stress condition. In order to establish the specific role of *SchSDD1-like* in stomatal abundance, we checked whether its overexpression could rescue the phenotype of *sdd1-3* Arabidopsis mutant plants. Typically, the epidermal phenotype of *sdd1-3* presented a combination of high stomatal abundance with some clustered stomata. All the *sdd1-3* transgenic lines overexpressing *SchSDD1-like* showed a significant reduction in leaf stomatal density compared to *sdd1-3* mutant plants. Furthermore, there were no detectable phenotypic abnormalities when compared with wild type plants, such as plant architecture, leaf size or shape. Interestingly, reduced stomatal density in the transgenic lines did not correlate with transgene transcript levels. A similar result was obtained when the overexpression of *AtSDD1* was conducted in *sdd1-1* mutant plants (Von Groll et al., 2002). The reduction in stomatal density observed in *sdd1-3*-rescued lines and tomato plants overexpressing *SchSDD1-like* was highly correlated, with decreased *SPCH* and putative *SolycSPCH-like* expression, respectively (**Figure 8**). These findings are in agreement with the regulatory pathway of stomatal development proposed in Arabidopsis (reviewed by Blatt et al., 2017; Chater et al., 2017; Qu et al., 2017). Thus, our findings indicate that *SchSDD1-like* is an ortholog of *SDD1* from *A. thaliana* and from potato plants. Since the *S. chilense* protein shares a very high structural homology with SolycSDD1-like, the gene that encodes this protein could be an ortholog of *AtSDD1* in cultivated tomato plants.

The fact that the expression level of *SchSDD1* is not directly proportional to the stomatal abundance in Arabidopsis and transgenic tomato plants (**Figures 4, 6**) could have different explanations depending on the proposed mechanism of SDD1 action in the pathway restricting stomatal development. For instance, the effect of ligand-receptor repression could be a result of saturation kinetics (Hulme and Trevethick, 2010). In this case, the recognition of the putative SDD1-borne ligand by TMM/ERf receptors may be saturated, even in transgenic plants with lower levels of *SDD1-like* overexpression. In addition, the availability of the SDD1-like substrate, rather than that of the subtilase itself, may be the limiting factor; in this case, a higher level of *SDD1-like* expression would have no greater effect on stomatal density. To ascertain this, a possible experimental approach could be to increase the availability of TMM/ERf receptors, together with *SchSDD1*, in transgenic lines of Arabidopsis or tomato plants.

Plants dissipate about 90% of their water content through the stomata by evapotranspiration (Schroeder et al., 2001). The water loss rate has been used as an important parameter for the hydric status of plants and as a reliable indicator of water deficit tolerance (Ganji Arjenaki et al., 2012). In the dehydration shock assay, we showed that *sdd1-3*-overexpressing *SchSDD1* increased water retention efficiency in a similar way to wild type plants, thus reverting the mutant phenotype. Several reports have established a relationship between stomatal density and WUE in Arabidopsis. The overexpression of *STOMAGEN*, a positive regulator of stomatal abundance, increased 2–3 fold stomatal density and transpiration rate, leading to a 50% decrease in WUE as compared to wild type plants (Sugano et al., 2010; Tanaka et al., 2013). Mutations in the transmembrane receptor ER (a negative regulator of stomatal abundance) also led to a decrease in WUE, which could be associated with enhanced stomatal density (Masle et al., 2005). Similar results have recently been reported concerning the upregulation of *YODA* (Meng and Yao, 2015). On the other hand, the repression of *AtSDD1* by GT2-like-1 (GTL1) can regulate WUE by modulating stomatal density (Yoo et al., 2010). Therefore, our findings confirm that low stomatal density can decrease the transpiration rate, thus improving WUE and consequently enhancing dehydration avoidance.

Transpiration, WUE and drought tolerance are the final result of a large number of processes. They are influenced by morphological leaf characteristics such as shape, thickness and internal structure, as well as by stomatal traits such as density, size, distribution across one or both epidermal sides, spacing patterns and degree of opening. Stomatal abundance is only one of the many traits that impinge on transpiration, and its correlation with WUE could be dependent on water availability and other external factors (Lawson et al., 2014). This array of behaviors shows the complexity inherent in determining the level of physiological output crucial for survival, such as the capacity to retain water. Adding to this complexity is the fact that many stomatal traits, both developmental and physiological, are subject to both environmental and genetic control (Hetherington and Woodward, 2003). On the other hand, environmental factors such as water or nutrient availability have a profound effect on stomatal size and density (Yan et al., 2012; Sun et al., 2014). In addition, genetics also play a role in determining these traits, either through natural variation (Delgado et al., 2011) or by directed gene modifications (reviewed by Franks et al., 2015).

Our results demonstrate that *SDD1-like* of *Solanum chilense* can regulate stomatal density and distribution in Arabidopsis, in a similar manner to *AtSDD1*. Furthermore, its overexpression in *S. lycopersicum* led to a decreased stomatal density and stomatal index on both sides of the leaf, as well as to improved dehydration avoidance in excised tomato leaves. These findings contribute to the knowledge of the molecular mechanisms involved in the regulation of stomatal development in two related tomato species, opening up possibilities for the investigation of the role of these genes and their expression patterns (*SchSDD1-like* and *SolycSDD1-like*) in the adaptation of plants to different environmental conditions, such as drought and salt stress.

## Author Contributions

SM-N, CF, and SR-L: conceived and designed the experiments. AO, AdM, and MM: performed confocal microscopy and determination of stomatal density analysis on leaves and cotyledons of wild type and transgenic Arabidopsis and tomato plants. SM-N, RP-D, and EG-V: performed expression analysis and functional complementation in Arabidopsis *sdd1-3* mutant and generation and analysis of tomato plants overexpressing SchSDD1-like. SM-N, MM, CF, and SR-L: prepared the manuscript.

## Conflict of Interest Statement

The authors declare that the research was conducted in the absence of any commercial or financial relationships that could be construed as a potential conflict of interest.

## Footnotes

1 solgenomics.net

2 http://www.megasoftware.net

3 www.ncbi.nlm.nih.gov/genbank/?

4 https://solgenomics.net/

5 www.gramene.org/

6 https://www.graphpad.com

7 https://solgenomics.net/organism/Solanum_lycopersicum/genome

## References

[B1] Berger D. Altmann T. (2000). A subtilisin-like serine protease involved in the regulation of stomatal density and distribution in *Arabidopsis thaliana*. *Genes Dev.* 14 1119–1131. 10.1101/gad.14.9.1119 10809670 PMC316574

[B2] Bergmann D. C. Sack F. D. (2007). Stomatal development. *Annu. Rev. Plant Biol.* 58 163–181. 10.1146/annurev.arplant.58.032806.104023 17201685

[B3] BHC (2004). *Modified Organism Potato Modified for Increased Stomata Density [Online].* Available at: https://bch.cbd.int/database/record.shtml?documentid=100293

[B4] Blatt M. R. Brodribb T. J. Torii K. U. (2017). Small pores with a big impact. *Plant Physiol.* 174 467–469. 10.1104/pp.17.00642 28584063 PMC5462035

[B5] Casson S. Gray J. (2008). Influence of environmental factors on stomatal development. *New Phytol.* 178 9–23. 10.1111/j.1469-8137.2007.02351.x 18266617

[B6] Cedzich A. Huttenlocher F. Kuhn B. M. Pfannstiel J. Gabler L. Stintzi A. (2009). The protease-associated domain and C-terminal extension are required for zymogen processing, Sorting within the secretory pathway, and activity of tomato Subtilase 3 (SlSBT3). *J. Biol. Chem.* 284 14068–14078. 10.1074/jbc.M900370200 19332543 PMC2682855

[B7] Chater C. C. C. Caine R. S. Fleming A. J. Gray J. E. (2017). Origins and evolution of stomatal development. *Plant Physiol.* 174 624–638. 10.1104/pp.17.00183 28356502 PMC5462063

[B8] Chen Z.-H. Chen G. Dai F. Wang Y. Hills A. Ruan Y.-L. (2017). Molecular evolution of grass stomata. *Trends Plant Sci.* 22 124–139. 10.1016/j.tplants.2016.09.005 27776931

[B9] Chou K.-C. Shen H.-B. (2010). Plant-mPLoc: a top-down strategy to augment the power for predicting plant protein subcellular localization. *PLoS One* 5:e11335. 10.1371/journal.pone.0011335 20596258 PMC2893129

[B10] Clauw P. Coppens F. De Beuf K. Dhondt S. Van Daele T. Maleux K. (2015). Leaf responses to mild drought stress in natural variants of Arabidopsis. *Plant Physiol.* 167 800–816. 10.1104/pp.114.254284 25604532 PMC4348775

[B11] Clough S. J. Bent A. F. (1998). Floral dip: a simplified method for Agrobacterium-mediated transformation of *Arabidopsis thaliana*. *Plant J.* 16 735–743. 10.1046/j.1365-313x.1998.00343.x 10069079

[B12] de Marcos A. Houbaert A. Triviño M. Delgado D. Martín-Trillo M. Russinova E. (2017). A mutation in the bHLH domain of the SPCH transcription factor uncovers a BR-dependent mechanism for stomatal development. *Plant Physiol.* 174 823–842. 10.1104/pp.17.00615 28507175 PMC5462054

[B13] de Marcos A. Triviño M. Fenoll C. Mena M. (2016). Too many faces for TOO MANY MOUTHS? *New Phytol.* 210 779–785. 10.1111/nph.13827 26742543

[B14] Delgado D. Alonso-Blanco C. Fenoll C. Mena M. (2011). Natural variation in stomatal abundance of *Arabidopsis thaliana* includes cryptic diversity for different developmental processes. *Ann. Bot.* 107 1247–1258. 10.1093/aob/mcr060 21447490 PMC3101138

[B15] Delgado D. Ballesteros I. Mena M. Fenoll C. (2012). Roles of constitutive photomorphogenic 10 in Arabidopsis stomata development. *Plant Signal. Behav.* 7 990–993. 10.4161/psb.20995 22836493 PMC3474701

[B16] Dodson G. Wlodawer A. (1998). Catalytic triads and their relatives. *Trends Biochem. Sci.* 23 347–352. 10.1016/S0968-0004(98)01254-7 9787641

[B17] Dow G. J. Berry J. A. Bergmann D. C. (2014). The physiological importance of developmental mechanisms that enforce proper stomatal spacing in *Arabidopsis thaliana*. *New Phytol.* 201 1205–1217. 10.1111/nph.12586 24206523

[B18] Emanuelsson O. Brunak S. Von Heijne G. Nielsen H. (2007). Locating proteins in the cell using targetP, signalP and related tools. *Nat. Protoc.* 2 953–971. 10.1038/nprot.2007.131 17446895

[B19] Engineer C. B. Ghassemian M. Anderson J. C. Peck S. C. Hu H. Schroeder J. I. (2014). Carbonic anhydrases, EPF2 and a novel protease mediate CO(2) control of stomatal development. *Nature* 513 246–250. 10.1038/nature13452 25043023 PMC4274335

[B20] Fillatti J. J. Kiser J. Ronald R. Comai L. (1987). Efficient transfer of a glyphosate tolerance gene into tomato using a binary *Agrobacterium tumefaciens* vector. *Nat. Biotechnol.* 5 726–730. 10.1038/nbt0787-726

[B21] Franks P. J. Doheny-Adams T. W. Britton-Harper Z. J. Gray J. E. (2015). Increasing water-use efficiency directly through genetic manipulation of stomatal density. *New Phytol.* 207 188–195. 10.1111/nph.13347 25754246

[B22] Ganji Arjenaki F. Jabbari R. Morshedi A. (2012). Evaluation of drought stress on relative water content, chlorophyll content and mineral elements of wheat (*Triticum aestivum* L.) Varieties. *Int. J. Agric. Crop Sci.* 4 726–729.

[B23] Gay A. P. Hurd R. G. (1975). The Influence of ligth on stomatal development density in the tomato. *New Phytol.* 75 37–46. 10.1111/j.1469-8137.1975.tb01368.x

[B24] Gray J. E. Hetherington A. M. (2004). Plant development: yoda the stomatal switch. *Curr. Biol.* 14 R488–R490. 10.1016/j.cub.2004.06.019 15203025

[B25] Gray J. E. Holroyd G. H. Van Der Lee F. M. Bahrami A. R. Sijmons P. C. Woodward F. I. (2000). The HIC signalling pathway links CO2 perception to stomatal development. *Nature* 408 713–716. 10.1038/35047071 11130071

[B26] Hall T. A. (1999). BioEdit: a user-friendly biological sequence alignment editor and analysis program for Windows 95/98/NT. *Nucl. Acids Symp. Ser.* 41 95–98.

[B27] Hamanishi E. T. Thomas B. R. Campbell M. M. (2012). Drought induces alterations in the stomatal development program in Populus. *J. Exp. Bot.* 63 4959–4971. 10.1093/jxb/ers177 22760471 PMC3427991

[B28] Hepworth C. Doheny-Adams T. Hunt L. Cameron D. D. Gray J. E. (2015). Manipulating stomatal density enhances drought tolerance without deleterious effect on nutrient uptake. *New Phytol.* 208 336–341. 10.1111/nph.13598 26268722 PMC4973681

[B29] Hetherington A. Woodward F. (2003). The role of stomata in sensing and driving environmental change. *Nature* 424 901–908. 10.1038/nature01843 12931178

[B30] Hulme E. C. Trevethick M. A. (2010). Ligand binding assays at equilibrium: validation and interpretation. *Br. J. Pharmacol.* 161 1219–1237. 10.1111/j.1476-5381.2009.00604.x 20132208 PMC3000649

[B31] Hunt L. Bailey K. J. Gray J. E. (2010). The signalling peptide EPFL9 is a positive regulator of stomatal development. *New Phytol.* 186 609–614. 10.1111/j.1469-8137.2010.03200.x 20149115

[B32] Jordá L. Conejero V. Vera P. (2000). Characterization of P69E and P69F, two differentially regulated genes encoding new members of the Subtilisin-Like proteinase family from tomato plants. *Plant Physiol.* 122 67–74. 10.1104/pp.122.1.67 10631250 PMC58845

[B33] Kanaoka M. M. Pillitteri L. J. Fujii H. Yoshida Y. Bogenschutz N. L. Takabayashi J. (2008). SCREAM/ICE1 and SCREAM2 specify three cell-state transitional steps leading to Arabidopsis stomatal differentiation. *Plant Cell* 20 1775–1785. 10.1105/tpc.108.060848 18641265 PMC2518248

[B34] Kang C.-Y. Lian H.-L. Wang F.-F. Huang J.-R. Yang H.-Q. (2009). Cryptochromes, phytochromes, and COP1 regulate light-controlled stomatal development in Arabidopsis. *Plant Cell* 21 2624–2641. 10.1105/tpc.109.069765 19794114 PMC2768914

[B35] Kearns E. V. Assmann S. M. (1993). The guard cell-environment connection. *Plant Physiol.* 102 711–715. 10.1104/pp.102.3.711 12231859 PMC158840

[B36] Kondo T. Kajita R. Miyazaki A. Hokoyama M. Nakamura-Miura T. Mizuno S. (2009). Stomatal density is controlled by a mesophyll-derived signaling molecule. *Plant Cell Physiol.* 51 1–8. 10.1093/pcp/pcp180 20007289

[B37] Lai L. B. Nadeau J. A. Lucas J. Lee E.-K. Nakagawa T. Zhao L. (2005). The Arabidopsis R2R3 MYB proteins FOUR LIPS and MYB88 restrict divisions late in the stomatal cell lineage. *Plant Cell* 17 2754–2767. 10.1105/tpc.105.034116 16155180 PMC1242270

[B38] Lake J. A. Quick W. P. Beerling D. J. Woodward F. I. (2001). Plant development: signals from mature to new leaves. *Nature* 411 154–154. 10.1038/35075660 11346781

[B39] Lau O. S. Bergmann D. C. (2012). Stomatal development: a plant’s perspective on cell polarity, cell fate transitions and intercellular communication. *Development* 139 3683–3692. 10.1242/dev.080523 22991435 PMC3445305

[B40] Lawson S. S. Pijut P. M. Michler C. H. (2014). Comparison of Arabidopsis stomatal density mutants indicates variation in water stress responses and potential epistatic effects. *J. Plant Biol.* 57 162–173. 10.1007/s12374-014-0017-1

[B41] Liu Y. Qin L. Han L. Xiang Y. Zhao D. (2015). Overexpression of maize SDD1 (ZmSDD1) improves drought resistance in *Zea mays* L. by reducing stomatal density. *Plant Cell Tiss. Organ Cult.* 122 147–159. 10.1007/s11240-015-0757-8

[B42] Livak K. J. Schmittgen T. D. (2001). Analysis of relative gene expression data using real-time quantitative PCR and the 2-ΔΔCT method. *Methods* 25 402–408. 10.1006/meth.2001.1262 11846609

[B43] Loyola J. Verdugo I. González E. Casaretto J. A. Ruiz-Lara S. (2012). Plastidic isoprenoid biosynthesis in tomato: physiological and molecular analysis in genotypes resistant and sensitive to drought stress. *Plant Biol.* 14 149–156. 10.1111/j.1438-8677.2011.00465.x 21974688

[B44] MacAlister C. A. Bergmann D. C. (2011). Sequence and function of basic helix-loop-helix proteins required for stomatal development in Arabidopsis are deeply conserved in land plants. *Evol. Dev.* 13 182–192. 10.1111/j.1525-142X.2011.00468.x 21410874 PMC3139685

[B45] MacAlister C. A. Ohashi-Ito K. Bergmann D. C. (2007). Transcription factor control of asymmetric cell divisions that establish the stomatal lineage. *Nature* 445 537–540. 10.1038/nature05491 17183265

[B46] Masle J. Gilmore S. R. Farquhar G. D. (2005). The ERECTA gene regulates plant transpiration efficiency in Arabidopsis. *Nature* 436 866–870. 10.1038/nature03835 16007076

[B47] Meichtry J. Amrhein N. Schaller A. (1999). Characterization of the subtilase gene family in tomato (*Lycopersicon esculentum* Mill.). *Plant Mol. Biol.* 39 749–760. 10.1023/A:1006193414434 10350089

[B48] Meng L.-S. Yao S.-Q. (2015). Transcription co-activator Arabidopsis ANGUSTIFOLIA3 (AN3) regulates water-use efficiency and drought tolerance by modulating stomatal density and improving root architecture by the transrepression of YODA (YDA). *Plant Biotechnol. J.* 13 893–902. 10.1111/pbi.12324 25599980

[B49] Meng X. Chen X. Mang H. Liu C. Yu X. Gao X. (2015). Differential function of Arabidopsis SERK family receptor-like kinases in stomatal patterning. *Curr. Biol.* 25 2361–2372. 10.1016/j.cub.2015.07.068 26320950 PMC4714584

[B50] Mizuno K. Nakamura T. Ohshima T. Tanaka S. Matsuo H. (1988). Yeast KEX2 gene encodes an endopeptidase homologous to subtilisin-like serine proteases. *Biochem. Biophys. Res. Commun.* 156 246–254. 10.1016/S0006-291X(88)80832-5 2845974

[B51] Orellana S. Yañez M. Espinoza A. Verdugo I. González E. Ruiz-Lara S. (2010). The transcription factor SlAREB1 confers drought, salt stress tolerance and regulates biotic and abiotic stress-related genes in tomato. *Plant Cell Environ.* 33 2191–2208. 10.1111/j.1365-3040.2010.02220.x 20807374

[B52] Peterson K. M. Rychel A. L. Torii K. U. (2010). Out of the mouths of plants: the molecular basis of the evolution and diversity of stomatal development. *Plant Cell* 22 296–306. 10.1105/tpc.109.072777 20179138 PMC2845417

[B53] Pillitteri L. J. Dong J. (2013). Stomatal development in Arabidopsis. *Arabidopsis Book* 11:e0162. 10.1199/tab.0162 23864836 PMC3711358

[B54] Pillitteri L. J. Sloan D. B. Bogenschutz N. L. Torii K. U. (2007). Termination of asymmetric cell division and differentiation of stomata. *Nature* 445 501–505. 10.1038/nature05467 17183267

[B55] Pillitteri L. J. Torii K. U. (2007). Breaking the silence: three bHLH proteins direct cell-fate decisions during stomatal development. *Bioessays* 29 861–870. 10.1002/bies.20625 17691100

[B56] Pillitteri L. J. Torii K. U. (2012). Mechanisms of stomatal development. *Annu. Rev. Plant Biol.* 63 591–614. 10.1146/annurev-arplant-042811-105451 22404473

[B57] Polgar L. (2005). The catalytic triad of serine peptidases. *Cell Mol. Life Sci.* 62 2161–2172. 10.1007/s00018-005-5160-x 16003488 PMC11139141

[B58] Popescu S. C. Popescu G. V. Bachan S. Zhang Z. Gerstein M. Snyder M. (2009). MAPK target networks in *Arabidopsis thaliana* revealed using functional protein microarrays. *Genes Dev.* 23 80–92. 10.1101/gad.1740009 19095804 PMC2632172

[B59] Qu X. Peterson K. M. Torii K. U. (2017). Stomatal development in time: the past and the future. *Curr. Opin. Genet. Dev.* 45 1–9. 10.1016/j.gde.2017.02.001 28219014

[B60] Raissig M. T. Matos J. L. Anleu Gil M. X. Kornfeld A. Bettadapur A. Abrash E. (2017). Mobile MUTE specifies subsidiary cells to build physiologically improved grass stomata. *Science* 355 1215–1218. 10.1126/science.aal3254 28302860

[B61] Rautengarten C. Steinhauser D. Büssis D. Stintzi A. Schaller A. Kopka J. (2005). Inferring hypotheses on functional relationships of genes: analysis of the *Arabidopsis thaliana* Subtilase gene family. *PLoS Comput. Biol.* 1:e40. 10.1371/journal.pcbi.0010040 16193095 PMC1236819

[B62] Rawlings N. D. Barrett A. J. Bateman A. (2010). MEROPS: the peptidase database. *Nucleic Acids Res.* 38 D227–D233. 10.1093/nar/gkp971 19892822 PMC2808883

[B63] Remans T. Smeets K. Opdenakker K. Mathijsen D. Vangronsveld J. Cuypers A. (2008). Normalisation of real-time RT-PCR gene expression measurements in *Arabidopsis thaliana* exposed to increased metal concentrations. *Planta* 227 1343–1349. 10.1007/s00425-008-0706-4 18273637

[B64] Ribeiro A. Akkermans A. D. van Kammen A. Bisseling T. Pawlowski K. (1995). A nodule-specific gene encoding a subtilisin-like protease is expressed in early stages of actinorhizal nodule development. *Plant Cell* 7 785–794. 10.1105/tpc.7.6.785 7647567 PMC160833

[B65] Riggs C. D. Zeman K. Deguzman R. Rzepczyk A. Taylor A. A. (2001). Antisense inhibition of a tomato meiotic proteinase suggests functional redundancy of proteinases during microsporogenesis. *Genome* 44 644–650. 10.1007/s00425-008-0706-4 11550900

[B66] Rose R. Schaller A. Ottmann C. (2010). Structural features of plant subtilases. *Plant Signal. Behav.* 5 180–183. 10.4161/psb.5.2.11069 20173418 PMC2884129

[B67] Sambrook J. Russell R. W. (2001). *Molecular Cloning: A Laboratory Manual* 3rd Edn. Cold Spring Harbor, NY: Cold Spring Harbor Laboratory Press.

[B68] Schaller A. Stintzi A. Graff L. (2011). Subtilases – versatile tools for protein turnover, plant development, and interactions with the environment. *Physiol. Plant.* 145 52–66. 10.1111/j.1399-3054.2011.01529.x 21988125

[B69] Schlüter U. Muschak M. Berger D. Altman T. (2003). Photosynthetic performance of an Arabidopsis mutant with elevated stomatal density (sdd1-1) under different light regimes. *J. Exp. Bot.* 54 867–874. 10.1093/jxb/erg087 12554730

[B70] Schroeder J. Kwak J. Allen G. (2001). Guard cell abscisic acid signalling and engineering drought hardiness in plants. *Nature* 410 327–330. 10.1038/35066500 11268200

[B71] Serna L. (2009). Cell fate transitions during stomatal development. *Bioessays* 31 865–873. 10.1002/bies.200800231 19565615

[B72] Shpak E. Mcabee J. Pillitteri L. Torii K. (2005). Stomatal patterning and differentiation by synergistic interactions of receptor kinases. *Science* 309 290–293. 10.1126/science.1109710 16002616

[B73] Smeekens S. P. Steiner D. F. (1990). Identification of a human insulinoma cDNA encoding a novel mammalian protein structurally related to the yeast dibasic processing protease Kex2. *J. Biol. Chem.* 265 2997–3000. 2154467

[B74] Sugano S. S. Shimada T. Imai Y. Okawa K. Tamai A. Mori M. (2010). Stomagen positively regulates stomatal density in Arabidopsis. *Nature* 463 241–244. 10.1038/nature08682 20010603

[B75] Sun Y. Yan F. Cui X. Liu F. (2014). Plasticity in stomatal size and density of potato leaves under different irrigation and phosphorus regimes. *J. Plant Physiol.* 171 1248–1255. 10.1016/j.jplph.2014.06.002 25014260

[B76] Tamura K. Stecher G. Peterson D. Filipski A. Kumar S. (2013). MEGA6: molecular evolutionary genetics analysis version 6.0. *Mol. Biol. Evol.* 30 2725–2729. 10.1093/molbev/mst197 24132122 PMC3840312

[B77] Tanaka Y. Sugano S. S. Shimada T. Hara-Nishimura I. (2013). Enhancement of leaf photosynthetic capacity through increased stomatal density in Arabidopsis. *New Phytol.* 198 757–764. 10.1111/nph.12186 23432385

[B78] Tapia G. Méndez J. Inostroza L. (2015). Different combinations of morpho-physiological traits are responsible for tolerance to drought in wild tomatoes *Solanum chilense* and *Solanum peruvianum*. *Plant Biol.* 18 406–416. 10.1111/plb.12409 26499789

[B79] Tapia G. Verdugo I. Yañez M. Ahumada I. Theoduloz C. Cordero C. (2005). Involvement of ethylene in stress-induced expression of the TLC1.1 retrotransposon from *Lycopersicon chilense* Dun. *Plant Physiol.* 138 2075–2086. 10.1104/pp.105.059766 16040666 PMC1183396

[B80] Tay A.-C. Furukawa A. (2008). Variations in leaf stomatal density and distribution of 53 vine species in Japan. *Plant Species Biol.* 23 2–8. 10.1111/j.1442-1984.2008.00201.x

[B81] Tomato Genome Consortium (2012). The tomato genome sequence provides insights into fleshy fruit evolution. *Nature* 485 635–641. 10.1038/nature11119 22660326 PMC3378239

[B82] Torii K. U. (2015). Stomatal differentiation: the beginning and the end. *Curr. Opin. Plant Biol.* 28 16–22. 10.1016/j.pbi.2015.08.005 26344486

[B83] Tornero P. Conejero V. Vera P. (1996). Primary structure and expression of a pathogen-induced protease (PR-P69) in tomato plants: similarity of functional domains to subtilisin-like endoproteases. *Proc. Natl. Acad. Sci. U.S.A.* 93 6332–6337. 10.1073/pnas.93.13.6332 8692815 PMC39022

[B84] Tricker P. J. Gibbings J. G. Rodriguez Lopez C. M. Hadley P. Wilkinson M. J. (2012). Low relative humidity triggers RNA-directed de novo DNA methylation and suppression of genes controlling stomatal development. *J. Exp. Bot.* 63 3799–3813. 10.1093/jxb/ers076 22442411 PMC3733579

[B85] Triviño M. Martín-Trillo M. Ballesteros I. Delgado D. De Marcos A. Desvoyes B. (2013). Timely expression of the Arabidopsis stoma-fate master regulator MUTE is required for specification of other epidermal cell types. *Plant J.* 75 808–822. 10.1111/tpj.12244 23662679

[B86] Vartapetian A. B. Tuzhikov A. I. Chichkova N. V. Taliansky M. Wolpert T. J. (2011). A plant alternative to animal caspases: subtilisin-like proteases. *Cell Death Differ.* 18 1289–1297. 10.1038/cdd.2011.49 21546909 PMC3172098

[B87] Villagarcia H. Morin A.-C. Shpak E. D. Khodakovskaya M. V. (2012). Modification of tomato growth by expression of truncated ERECTA protein from *Arabidopsis thaliana*. *J. Exp. Bot.* 63 6493–6504. 10.1093/jxb/ers305 23096000

[B88] Von Groll U. Berger D. Altmann T. (2002). The Subtilisin-like serine protease SDD1 mediates cell-to-cell signaling during Arabidopsis stomatal development. *Plant Cell* 14 1527–1539. 10.1105/tpc.001016 12119372 PMC150704

[B89] Vrablova M. Vrabl D. Hronkova M. Kubasek J. Santrucek J. (2017). Stomatal function, density and pattern, and CO2 assimilation in *Arabidopsis thaliana* tmm1 and sdd1-1 mutants. *Plant Biol.* 19 689–701. 10.1111/plb.12577 28453883

[B90] Wang C. Liu S. Dong Y. Zhao Y. Geng A. Xia X. (2016). PdEPF1 regulates water-use efficiency and drought tolerance by modulating stomatal density in poplar. *Plant Biotechnol. J.* 14 849–860. 10.1111/pbi.12434 26228739 PMC11388919

[B91] Wang H. Ngwenyama N. Liu Y. Walker J. C. Zhang S. (2007). Stomatal development and patterning are regulated by environmentally responsive mitogen-activated protein kinases in Arabidopsis. *Plant Cell* 19 63–73. 10.1105/tpc.106.048298 17259259 PMC1820971

[B92] Warnock S. J. (1991). Natural habitats of Lycopersicon species. *Hortscience* 26 466–471.

[B93] Wells J. A. Ferrari E. Henner D. J. Estell D. A. Chen E. Y. (1983). Cloning, sequencing, and secretion of *Bacillus amyloliquefaciens* subtilisin in *Bacillus subtilis*. *Nucleic Acids Res.* 11 7911–7925. 10.1093/nar/11.22.7911 6316278 PMC326549

[B94] Wise R. J. Barr P. J. Wong P. A. Kiefer M. C. Brake A. J. Kaufman R. J. (1990). Expression of a human proprotein processing enzyme: correct cleavage of the von Willebrand factor precursor at a paired basic amino acid site. *Proc. Natl. Acad. Sci. U.S.A.* 87 9378–9382. 10.1073/pnas.87.23.9378 2251280 PMC55168

[B95] Xiao Y. Yu X. Chen J. Di P. Chen W. Zhang L. (2009). IiSDD1, a gene responsive to autopolyploidy and environmental factors in Isatis indigotica. *Mol. Biol. Rep.* 37 987–994. 10.1007/s11033-009-9776-z 19728150

[B96] Xie C. Zhang R. Qu Y. Miao Z. Zhang Y. Shen X. (2012). Overexpression of MtCAS31 enhances drought tolerance in transgenic Arabidopsis by reducing stomatal density. *New Phytol.* 195 124–135. 10.1111/j.1469-8137.2012.04136.x 22510066

[B97] Xu Z. Zhou G. (2008). Responses of leaf stomatal density to water status and its relationship with photosynthesis in a grass. *J. Exp. Bot.* 59 3317–3325. 10.1093/jxb/ern185 18648104 PMC2529243

[B98] Yamagata H. Masuzawa T. Nagaoka Y. Ohnishi T. Iwasaki T. (1994). Cucumisin, a serine protease from melon fruits, shares structural homology with subtilisin and is generated from a large precursor. *J. Biol. Chem.* 269 32725–32731. 7806492

[B99] Yan F. Sun Y. Song F. Liu F. (2012). Differential responses of stomatal morphology to partial root-zone drying and deficit irrigation in potato leaves under varied nitrogen rates. *Sci. Hortic.* 145 76–83. 10.1016/j.scienta.2012.07.026

[B100] Yang M. Sack F. D. (1995). The too many mouths and four lips mutations affect stomatal production in Arabidopsis. *Plant Cell* 7 2227–2239. 10.1105/tpc.7.12.2227 11536724 PMC161075

[B101] Yoo C. Y. Pence H. E. Jin J. B. Miura K. Gosney M. J. Hasegawa P. M. (2010). The Arabidopsis GTL1 transcription factor regulates water use efficiency and drought tolerance by modulating stomatal density via transrepression of SDD1. *Plant Cell* 22 4128–4141. 10.1105/tpc.110.078691 21169508 PMC3027182

