# Supplementary material for: Overexpression of a SDD1-Like Gene From Wild Tomato Decreases Stomatal Density and Enhances Dehydration Avoidance in Arabidopsis and Cultivated Tomato
Source: Front Plant Sci. 2018 Jul 4;9:940. doi: 10.3389/fpls.2018.00940 (PMC6039981; doi:10.3389/fpls.2018.00940)
Supplement: Supplementary file 1 [file Table_1.PDF]

Supplementary Table S1: Database and accession numbers of the SBTs selected to draw the phylogenetic tree

Solanum lycopersicum	SolycSDD1-like (Solyc09g064490)[a]	SolycSBT2 (CAA64730)[c]	SolycP69A (CAA76724)[c]	SolycSBT1 (CAA67429)[c]	SolycSBT3 (NP_001234774)[c]	SolycTMP (AAB38743)[c]	SolycSBT4a (NP_001234780)[c]
Solanum pennellii	SopenSDD1-like (Sopen09g025640)[a]						
Solanum tuberosum	StuSDD1 (PGSC0003DMT400085430)[e]						
Vitis vinifera	VvSDD1 (VIT_07s0005g04410)[e]						
Prunus persica	PperSDD1 (EMJ03000)[c]						
Populus trichocarpa	PtrSDD1-like (POPTR_0002s25800)[e]						
Isastis tinctoria L	ItSDD1 (ABD64827)[c]						
Arabidopsis thaliana	AtSDD1 (NP_563701)[c]	AtAIR3 (AAK74005)[c]	At1g32960 (NP_568255)[c]	AtXSP1 (NP_568889)[c]	AtALE1 (NP_564793)[c]	AtS1P (AAM97020)[c]	
Oryza sativa	OsSDD1-like (BGIOSGA011836-PA)[f]						
Zea mays	ZmSDD1-like (GMZM2G107686_P01)[d]						
Nicotiana benthamiana	NbSDD1-like (NbS00008079g0002)[a]						
Sorghum bicolor	SbSDD1-like (SBo01g047390)[d]						
Solanum chilense	SchSBT2-like						
Triticum aestivum	TaSDD1-like (Traes_2DL_6B5F4F3CF)[b]						
Cucumis melo	pre-pro-cucumisin (BAA06905)[c]						
Glycine max	GmSDD1-like (Glyma036324701)[d]						
Medicago truncatula	SDD1-like (AES81608)[c]						
Brassica oleracea	BoSDD1 (Bo5g004320)[e]						

Upper letter means source database: a, Solgenomics; b, Phytozome; c, GenBank; d, PlantgDB; e, Gramene; f, BGI RiceDB.
