# Supplementary material for: Overexpression of a SDD1-Like Gene From Wild Tomato Decreases Stomatal Density and Enhances Dehydration Avoidance in Arabidopsis and Cultivated Tomato
Source: Front Plant Sci. 2018 Jul 4;9:940. doi: 10.3389/fpls.2018.00940 (PMC6039981; doi:10.3389/fpls.2018.00940)
Supplement: Supplementary file 2 [file Image_1.PDF]

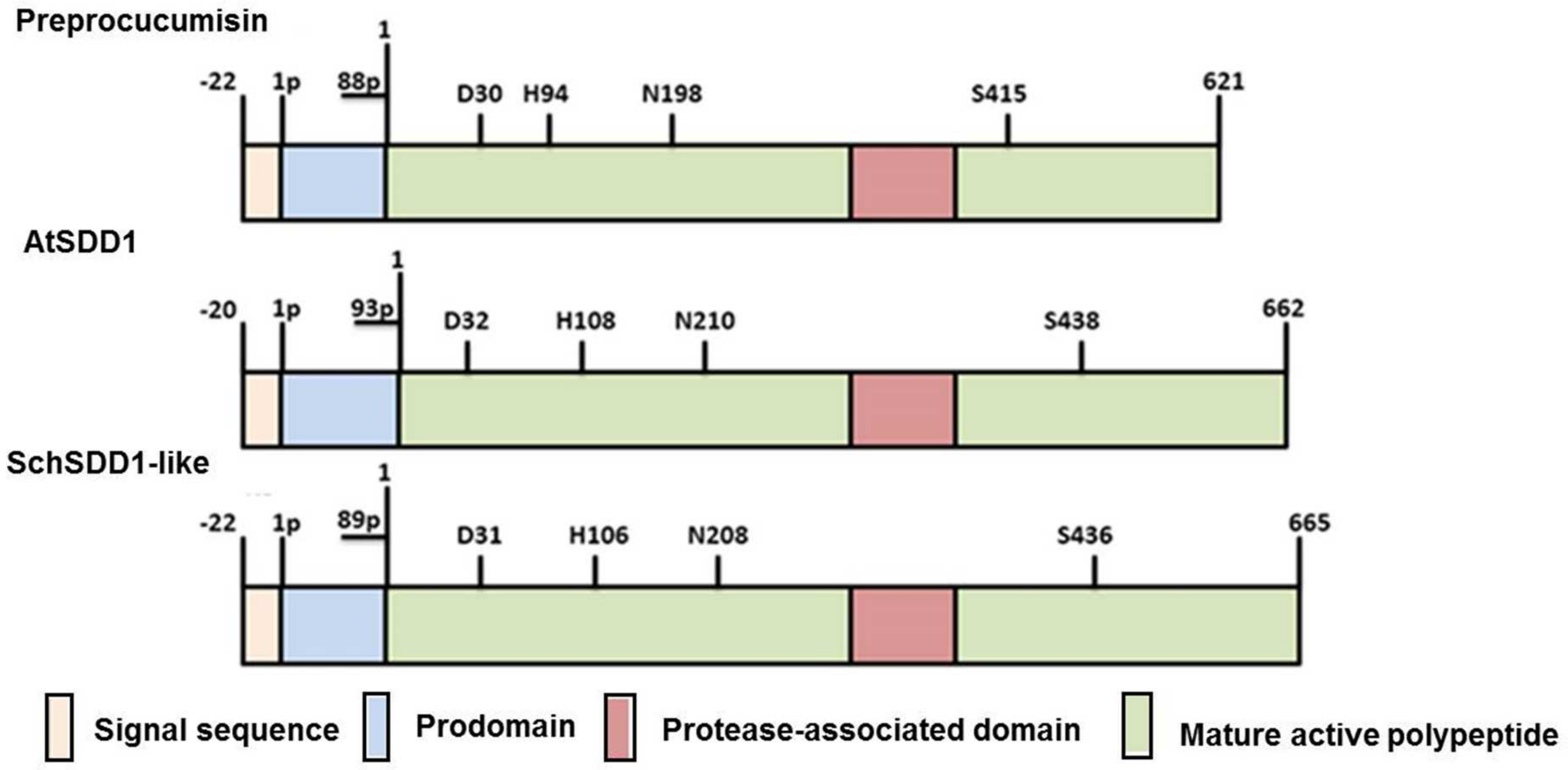

Supplementary Figure S1. Schematic representation of preproprotein structures for the two best-known members of the subtilisin-like plant family and SchSDD1. Signal sequences are indicated by negative numbers, while each prodomain is indicated using numbers followed by ‘‘p’’. The numbering of the mature enzyme indicates the positions of the N and C termini. Residues of the catalytic triad, i.e., Asp, His and Ser, are labelled, as is a conserved Asn involved in oxyanion hole stabilization. The core conserved region of the protease-associated (PA) domain, which is predicted to be involved in protease-substrate interaction (Mahon and Bateman, 2000), is also indicated. The scheme is based as described by Beers *et al.* (2004).
