# Supplementary material for: Overexpression of a SDD1-Like Gene From Wild Tomato Decreases Stomatal Density and Enhances Dehydration Avoidance in Arabidopsis and Cultivated Tomato
Source: Front Plant Sci. 2018 Jul 4;9:940. doi: 10.3389/fpls.2018.00940 (PMC6039981; doi:10.3389/fpls.2018.00940)
Supplement: Supplementary file 3 [file Image_2.PDF]

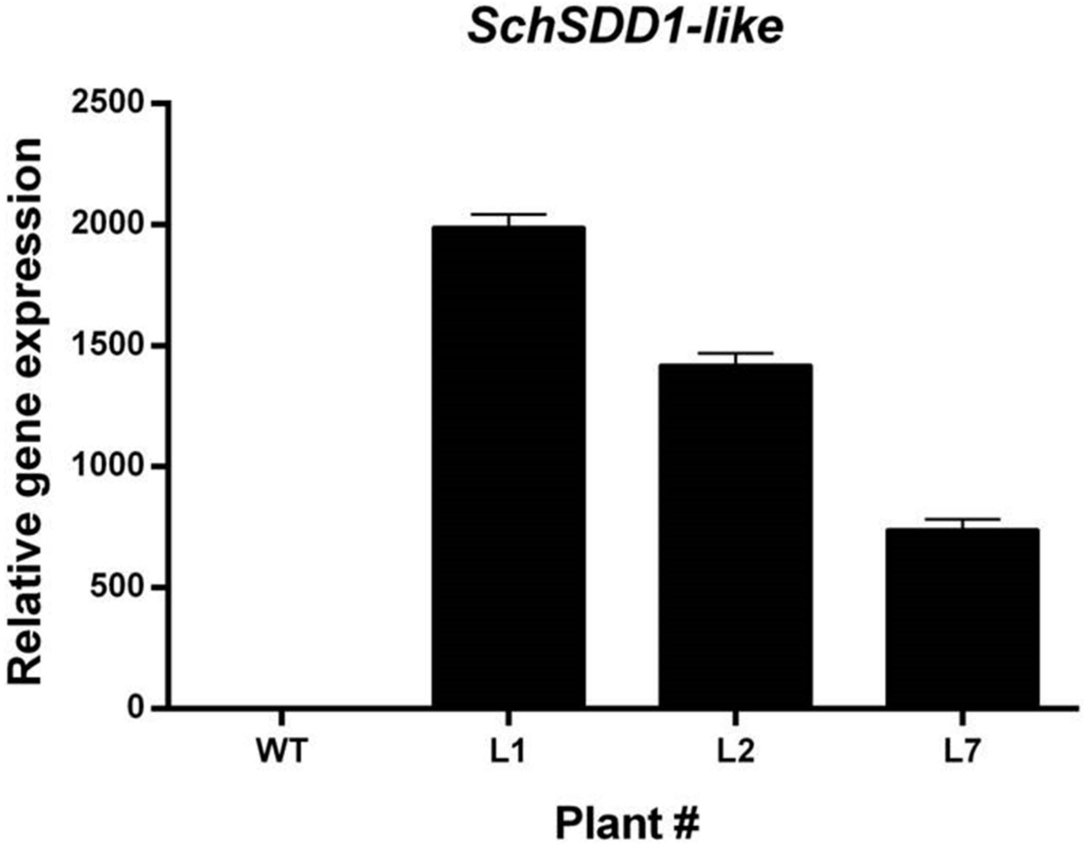

Supplementary Figure S2. Ectopic gene expression of *SDD1-like* isolated from *S. chilense* in cultivated tomato plants. The transcript levels of RNA extracted from the leaves of wild type and transgenic plants were evaluated by qPCR. The relative level of expression was measured using *SolycGAPDH* as a normalizer gene; the values shown correspond to mean ± standard error from three biological replicates.
