# Supplementary material for: Overexpression of a SDD1-Like Gene From Wild Tomato Decreases Stomatal Density and Enhances Dehydration Avoidance in Arabidopsis and Cultivated Tomato
Source: Front Plant Sci. 2018 Jul 4;9:940. doi: 10.3389/fpls.2018.00940 (PMC6039981; doi:10.3389/fpls.2018.00940)
Supplement: Supplementary file 4 [file Image_3.PDF]

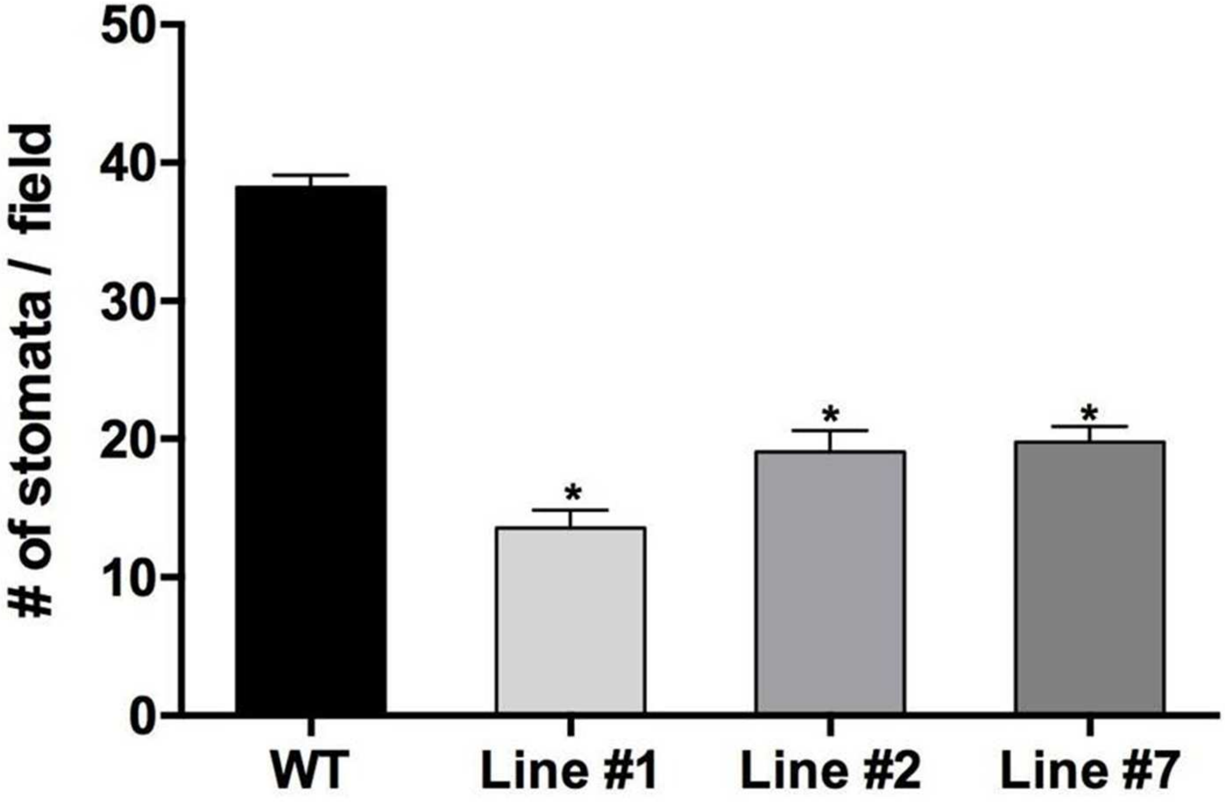

Supplementary Figure S3. Stomatal frequency (number of stomata per field) in *S. lycopersicum* (cv. Moneymaker) and *SchSDD1-like*-overexpressing tomato plants. Stomatal quantification was performed on the abaxial epidermis of fully expanded leaves. The values correspond to the mean of values of four biological replicates. The asterisk symbol * shows significant differences between wild type and transgenic plants (Student's t-test, $p<0.05$).
